# Testing the psychometric properties of the illness perceptions questionnaire for OCD (IPQ-O)

**DOI:** 10.1186/s12888-019-2195-3

**Published:** 2019-07-10

**Authors:** Rebecca Pedley, Katherine Berry, Penny Bee, Judith Gellatly, Alison Wearden

**Affiliations:** 1 Division of Psychology and Mental Health, School of Health Sciences, Faculty of Biology, Medicine and Health, Manchester Academic Health Science Centre, The University of Manchester, Oxford Road, Manchester, M13 9PL UK; 2 Division of Nursing, Midwifery and Social Work, School of Health Sciences, Faculty of Biology, Medicine and Health, Manchester Academic Health Science Centre, The University of Manchester, Oxford Road, Manchester, M13 9PL UK; 3 Division of Psychology and Mental Health, School of Health Sciences & Manchester Centre for Health Psychology, Faculty of Biology, Medicine and Health, Manchester Academic Health Science Centre, The University of Manchester, Oxford Road, Manchester, M13 9PL UK

**Keywords:** Obsessive-compulsive disorder, Illness perceptions, Common-sense model, Questionnaire, Psychometrics

## Abstract

**Background:**

Previous research has shown that our perceptions about illness are important determinants of how we respond and adjust to health threats. To examine whether illness perceptions affect illness responses in OCD (e.g. help-seeking), this study aimed to develop and test the psychometric properties of a new OCD-specific tool to assess illness perceptions, the illness perceptions questionnaire for OCD (IPQ-O).

**Methods:**

A cross-sectional questionnaire-based design was used. Following adaptation of the IPQ-R based on qualitative interviews with people with OCD, adults (age ≥ 16) with OCD completed the IPQ-O (online or postal), alongside measures of depression, anxiety, OCD severity, attitudes to seeking mental health services and behaviours (e.g. treatment seeking intentions). A sub-sample re-completed the IPQ-O after two-weeks to obtain test-retest reliability. Factor analysis was used to derive the IPQ-O factor structure; internal consistency of subscales was calculated. Convergent validity was explored.

**Results:**

Three hundred forty-eight people with OCD completed the IPQ-O. After factor analysis, seven main sub-scales and four cause sub-scales were identified, explaining 45.5 and 41.6% of the variance after extraction and rotation respectively. Three sub-scales from the original IPQ-R were validated; other dimensions differed from original IPQ-R sub-scales. The new ‘spectrum’ sub-scale measures the strength of the view that OCD is a trait that presents to varying extents within the general population. The IPQ-O demonstrated internal consistency, test re-test reliability (Kendall’s tau = .51–.75) and convergent validity. Illness perceptions were associated with important aspects of adjustment (depression, anxiety) and condition management (receipt of treatment, plans to seek help). In particular, emerging data showed that those who had not received medication for OCD endorsed stronger spectrum beliefs. Though longitudinal study is needed to verify the direction of this association, this raises the question of whether spectrum beliefs deter people with OCD from using pharmacological treatments.

**Conclusions:**

The IPQ-O provides a valuable tool for subsequent testing of whether illness perceptions drive outcomes as proposed by the CSM. If perceptions are found to drive adjustment and behaviour, therapists could elicit and subsequently challenge perceptions that have negative effects on adjustment and coping, as part of psychological therapy.

## Background

The common-sense model (CSM) proposes that our personal understandings of illness are key determinants of how we respond and adjust to health threats [1, 2]. The validity of the CSM has been widely tested through a large body of research in physical health conditions. The development of the Illness perceptions questionnaire (IPQ), and its revised form the IPQ-R, has contributed enormously to this research as these measures enable perceptions about illness to be quantifiably assessed [3, 4]. The IPQ-R assesses two key components of illness understanding posited within the CSM; cognitive and emotional representations of illness, together known as illness perceptions [4]. In the IPQ-R, the main section consists of seven sets of Likert-type scales, which are summed to measure six dimensions of cognitive representations (timeline acute/chronic, consequences, personal control, treatment control, illness coherence, and timeline cyclical) and one emotional representation dimension. The remaining two dimensions of cognitive representations described in the CSM, identity and cause, are measured separately through two individual scales. A meta-analysis of 45 studies testing the CSM in physical health conditions found that illness perceptions were associated with key illness responses and outcomes, such as coping strategies and levels of psychological distress [5].

A growing body of literature has sought to understand the CSM’s utility in mental health conditions. A systematic review found that the dimensions of cognitive and emotional representations applied to mental health conditions and that, as with physical health conditions, these perceptions link to variance in personal response and outcomes [6]. On the other hand, several qualitative studies have cast doubt on the application of the CSM to mental health conditions, for example, in the case of a study on postnatal depression, the authors concluded that understandings of the condition were too complex to fit within ‘neat’ dimensions of understanding [7]. Baines and Wittkowski (6) highlight the need for further qualitative exploration of illness perceptions in mental health, to identify potential, additional categories of perception and to facilitate the adaptation of tools such as the IPQ-R.

Obsessive-compulsive disorder (OCD) is a debilitating mental health condition, with a frequently chronic course and impairing effect on sufferers of the condition [8] and their family members alike [9]. There is a substantial average gap of up to 17 years between symptom onset and receipt of treatment [10, 11], together with high rates of disengagement from efficacious treatments such as psychological therapy [12]. To our knowledge, no study has tested the associations between illness perceptions and these behavioural responses in OCD.

The primary aim of this study was to test the psychometrics of a new version of the IPQ-R, which has been adapted for OCD following preliminary qualitative work [13]. A secondary aim was to examine associations between perceptions of OCD with emotional responses, help-seeking intentions and treatment use. Understanding the relationships between perceptions of OCD and these responses would both provide support for the CSM and suggest ways in which condition management could be enhanced and psychological distress reduced.

## Methods

### Design

A cross-sectional design was used to assess the psychometric properties of the illness perceptions questionnaire for OCD (IPQ-O), including its dimensionality, internal consistency and convergent validity. Test re-test reliability was assessed using a two-week re-test.

Ethical approval for this study was given by the National Research Ethics Service Committee North West – Lancaster (reference number 16/NW/0050).

### Participants

Eligible participants were aged ≥16, with a self-reported diagnosis of OCD from a health professional and the ability to read and write in English. There were no exclusion criteria.

### Recruitment

Participants were recruited through advertisement and direct invitation. Advertisements were circulated through relevant UK charity websites, social media channels and OCD support groups. Questionnaire packs and study advertisements were also distributed at a national (UK) OCD charity conference. Participants who had taken part in a previous qualitative study and members of an OCD research register (*n* = 83) were invited by email or letter.

### Materials

Participants were asked for their basic demographic details and completed the following questionnaires in paper or electronic format:

#### OCD condition management questionnaire

Participants were asked for length of time living with OCD (years). Participants indicated their previous treatment use (e.g. medication, talking therapies) as well as help seeking intentions (e.g. plans to seek support from the NHS, private therapy etc.) by checking all applicable boxes.

#### The illness perceptions questionnaire for OCD (IPQ-O)

Potential items for the IPQ-O were generated from themes identified from qualitative analysis of 16 interviews with people with OCD [13]. From this analysis, three potential additional dimensions, not currently assessed in the IPQ-R, emerged: 1) perceptions of the condition as ‘part’ of the self, 2) perceptions of the reactivity of the condition to external and internal influences and 3) beliefs about OCD as a spectrum disorder within the general population.

As advocated by Moss-Morris et al. [4], the identity scale and cause scale of the IPQ-R were revised to ensure that the measure assessed symptoms and causes that people with OCD see as relevant to their condition. All items in the main-section scales of the IPQ-R were retained with minor amendments to wording. The final measure contained a total of 23 identity items, 17 cause items and 76 main sub-scale items. Of the main sub-scale items, 53 items represented existing dimensions of the IPQ-R and 23 items represented potential new sub-scales.

All items, except those contributing to the identity scale, are rated on a five-point Likert scale, ranging from strongly disagree to strongly agree. The order of the main section items was randomised prior to finalisation of the questionnaire; the cause items were presented in a separate section. In line with the IPQ-R, the identity scale followed a two-column format, whereby participants were first asked to answer ‘yes’ or ‘no’, firstly to whether a symptom had been experienced, and secondly, whether this symptom was ‘related’ to their condition.

To ensure acceptability and clarity of the IPQ-O, seven people with lived experience of OCD were asked to comment on the draft measure. Feedback indicated that the measure was understandable and straightforward to complete. Comments were used to make refinements, including defining symptoms in the identity list (e.g. ‘hypervigilance’), adding explanatory text to clarify that there are no ‘right or wrong’ answers and simplifying instructions.

#### The self-rated Yale-Brown obsessive compulsive scale (SR Y-BOCS) [14]

An adapted version of the original gold-standard clinician rated Y-BOCS, this 10-item self-rated measure assesses the severity of OCD symptoms experienced in the last week. It yields a total score of 0–40, with higher scores indicating greater severity.

#### The work and social adjustment scale (WSAS) [15]

This five-item self-report measure assesses functional impairment resulting from a clinical problem from the patient perspective. Scores range from 0 to 40, with lower scores reflecting better functioning. The measure has demonstrated reliability and validity in mental health samples, including OCD [15].

#### The Patient Health Questionnaire (PHQ-9) [16, 17]

With a total score range of 0–27 across nine items, with higher scores indicating greater severity. This self-report measure assesses depression in primary care populations, over the last two-weeks. The measure has good psychometric properties [16].

#### Generalised Anxiety Disorder 7 (GAD-7) [18]

Total scores on the seven-item scale range from 0 to 21 (higher scores indicating greater severity) based on patient ratings of their symptoms of generalised anxiety disorder (GAD) in the last two weeks. The measure has demonstrated reliability and validity [18].

#### Inventory of attitudes toward seeking mental health services (IASMHS) [19]

This 24-item measure assesses three sub-scales: psychological openness, help-seeking propensity and indifference to stigma. Each eight-item sub-scale is scored 0–32, scored using a five-point Likert scale ranging from disagree (0) to agree (4), with higher scores indicating more positive views.

### Statistical analysis

Analysis was conducted using IBM SPSS Statistics Version 23 [20].

#### Identity scale

Cases who had more than two missing data points were excluded from analyses that involved the identity scale. Descriptive statistics were calculated (proportion of individuals endorsing each symptom) to assess the validity of the symptoms in the identity list. Cronbach’s coefficient alpha (α) assessed the internal consistency of the scale.

#### Main sub-scales factor structure

In line with the approach taken by Moss-Morris et al. [4], the dimensional structure of items from the main set sub-scales was assessed. Items contributing to the potential three new sub-scales were entered into the analysis together with original IPQ-R sub-scale questions. A principal axis factor analysis was selected due to the non-normal distribution of data [21]. As it was expected that the resultant sub-scales would correlate, an oblique rotation (direct oblimin) was chosen [21].

Before any analysis was undertaken, reverse scored items were re-coded. Prior to factor analysis, it was necessary to manage missing data. Little’s test was non-significant, suggesting that data were missing completely at random (MCAR) and could therefore be appropriately handled through listwise deletion or simple imputation (*χ*^2^ = 4373.892, *df* = 4289, *P* = .179). To reduce the potential for unpredictable bias resulting from the substantial case loss incurred through list-wise deletion [22], missing data points were imputed through expectation-maximisation (EM).

The sample size exceeded 300 cases, suggesting that factor analysis would yield a stable solution [23]. Kaiser-Meyer-Olkin (KMO) statistics were calculated to verify that all items/scales met the minimum sampling adequacy criterion of ≥.5 [23, 24]. The correlation matrix was inspected to identify and subsequently remove any items which risked extreme multicollinearity (r ≥ .8) [23].

As the break in the scree plot was ambiguous, six, seven, and eight factors were tested to identify which number provided the cleanest and most interpretable structure [21]. To improve the interpretability of the structure further, items with weak loadings across all factors (≤.3) were removed and the analysis was re-run [25]. Any cross-loading items were allocated to the factor which they fitted with best in terms of their conceptual similarity [25]. Internal consistency was evaluated using Cronbach’s α, employing a minimum value of .7 to indicate adequate reliability [26]. Items which reduced or which did not contribute any additional value to sub-scale α were removed [25]. Additionally, Cronbach’s α was used as an item-reduction method to reduce sub-scales exceeding 15 items to the recommended range of 10–15 items [25]. Finally the subscales derived from the final factor solution were interpreted and named.

#### Cause scale factor structure

A separate principal axis factor analysis with direct oblimin rotation was conducted on the cause items. Little’s test was non-significant indicating that listwise deletion or simple imputation were appropriate methods of handling missing data (*χ*^**2**^ = 301.594, *df* = 270, *P* = .090). Missing data were imputed through EM. The remainder of the analysis followed the same methods outlined for the main sub-scales analysis.

#### Psychometric testing

To facilitate reliability and validity testing of the factor analysed scales (the main sub-scales and cause sub-scales), items representing each sub-scale were summed. Scores for the identity scale were calculated based on the total number of symptoms that the individual perceived as ‘related’ (i.e. the sum of ‘yes’ rated symptoms) to their OCD.

As the total sub-scale scores for items rated on a Likert scale were non-normally distributed, non-parametric tests were used to conduct reliability and validity analyses. Kendall’s Tau (τ) was selected for test-re-test correlations between baseline and two-week IPQ-O data, as well as for correlations between IPQ-O sub-scales and with other measures to assess construct validity (PHQ-9, GAD-7, IASMHS, help seeking and treatment receipt variables).

Commonly used criteria for interpreting correlation effect sizes .10 (small), .30 (medium) and .50 (large) [27] and adequate test-retest reliability (.7) [26] are based on Pearson’s correlation coefficient (*r*), which is often numerically larger than τ. A conversion table that facilitated transformation between τ and *r* [28] was used to provide equivalent values which could be used as ‘rules of thumb’ when interpreting the current findings: .06 (small), .19 (medium), .33 (large), acceptable test-re-test reliability >.49.

Mann-Whitney U tests were used to test for statistical differences in IPQ-O scores amongst categorical data, including whether or not individuals had accessed treatment, or planned to seek help.

### Procedure

Participants were provided with an information sheet and given the choice of online or postal participation. Eligibility was assessed by completion of a form, verifying age and confirming (through self-report) receipt of past OCD diagnosis by indicating ‘yes’ or ‘no’ to the following question: ‘Have you received a diagnosis of OCD from a health professional?’. Questionnaires were completed in the following order: OCD help-seeking questionnaire, IPQ-O, SR Y-BOCS, WSAS, PHQ-9, GAD-7, IASMHS. Following completion, participants were given the option to return the questionnaire anonymously, or if consenting to take part in a two-week re-test, to provide their contact details. Two-week re-test invites were sent until a minimum of 50 IPQ-O completions had been obtained.

Recruitment took place between 20.05.16 and 31.07.17. The option to participate in a prize draw to win a £50 shopping voucher was offered to all participants. All participants gave their informed consent for participation, as indicated by questionnaire return. This method of consent was used at the request of the approving ethics committee.

## Results

### Participants

A total of 348 participants provided some IPQ-O data, of which all participants provided identity scale data, 346 provided main sub-scale data and 331 completed cause scale data. The majority of individuals participated online (*n* = 343). Sample characteristics can be viewed in Table 1. Of the 111 people invited to re-take the IPQ-O, 64 took part, with all but one participating online.

**Table 1:** Demographic and clinical characteristics of sample at baseline (*n* = 348)

		Missing data (N)
Age (years) M, SD (range)	33.16, 12.06 (16-79)	19
Years experiencing OCD M, SD (range)	18.35, 12.75 (1-72)	10
Gender N (%)	Female = 262 (75.3%) Male = 82 (23.6%) Transgender = 1 (0.3%) Prefer not to answer = 2 (0.6%)	1
Past treatment for OCD received N (%)	No previous treatment received = 9 (2.3%) Cognitive Behaviour Therapy = 277 (79.6%) Other talking therapy = 109 (31.3%) Medication = 251 (72.1%) Other treatment = 42 (12.07%) *Total exceeds 100% as some individuals received more than one treatment*	2
Employment status N (%)	Employed: 193 (55.5%) Retired = 12 (3.4%) Looking after home or family = 21 (6.0%) Not employed = 41 (11.8%) Full time student = 54 (15.5%) Other/prefer not to answer: 26 (7.5%)	1

### Identity scale validity and internal reliability

Of the 348 participants providing identity data, 276 responses were included in the descriptive analyses of the identity scale, with the remaining individuals (*n* = 72) having missed two or more data-points. All identity scale symptoms were endorsed by a significant proportion of participants (Table 2), with compulsions constituting the most widely endorsed symptom (96%) and disorientation, the least (30.1%). The majority of symptoms were endorsed by over half the participants, supporting the validity of the scale. Cronbach’s α for the scale was .87 indicating a high level of internal consistency.

**Table 2:** Identity scale presented in order of symptoms mostly highly endorsed as ‘related’ to OCD (*n* = 272–276)

	N endorsing symptom	Percentage of respondents	Sample N
Compulsive behaviours or rituals (e.g. Handwashing, checking, counting silently to yourself, reassurance seeking)	265	96.0%	276
Obsessions (unpleasant thoughts, images or impulses that come into your mind repeatedly)	263	95.3%	274
Irrational thoughts	250	90.6%	276
Anxiety	244	88.4%	275
Feelings of unease	235	85.1%	275
Thinking too much about things	234	84.8%	276
Worrying too much	224	81.2%	276
Feeling tense	223	80.8%	276
Difficulty concentrating	209	75.7%	275
Low mood	206	74.6%	276
Always expecting the worst to happen	204	73.9%	275
Hypervigilance (constantly looking for danger, threats or harm)	199	72.1%	276
A fixed way of thinking	187	67.8%	276
Irritability	185	67.0%	275
Feeling withdrawn	174	63.0%	276
Restlessness	170	61.6%	273
Difficulty experiencing pleasure	169	61.2%	273
Paranoia	156	56.5%	273
Suicidal thoughts	154	55.8%	276
Lacking energy or motivation	148	53.6%	272
Sleep problems	146	52.9%	276
Feeling ‘dissociated’ or disconnected from yourself	136	49.3%	276
Disorientation	83	30.1%	273

### Factor structure and internal reliability

**Fig. 1:**
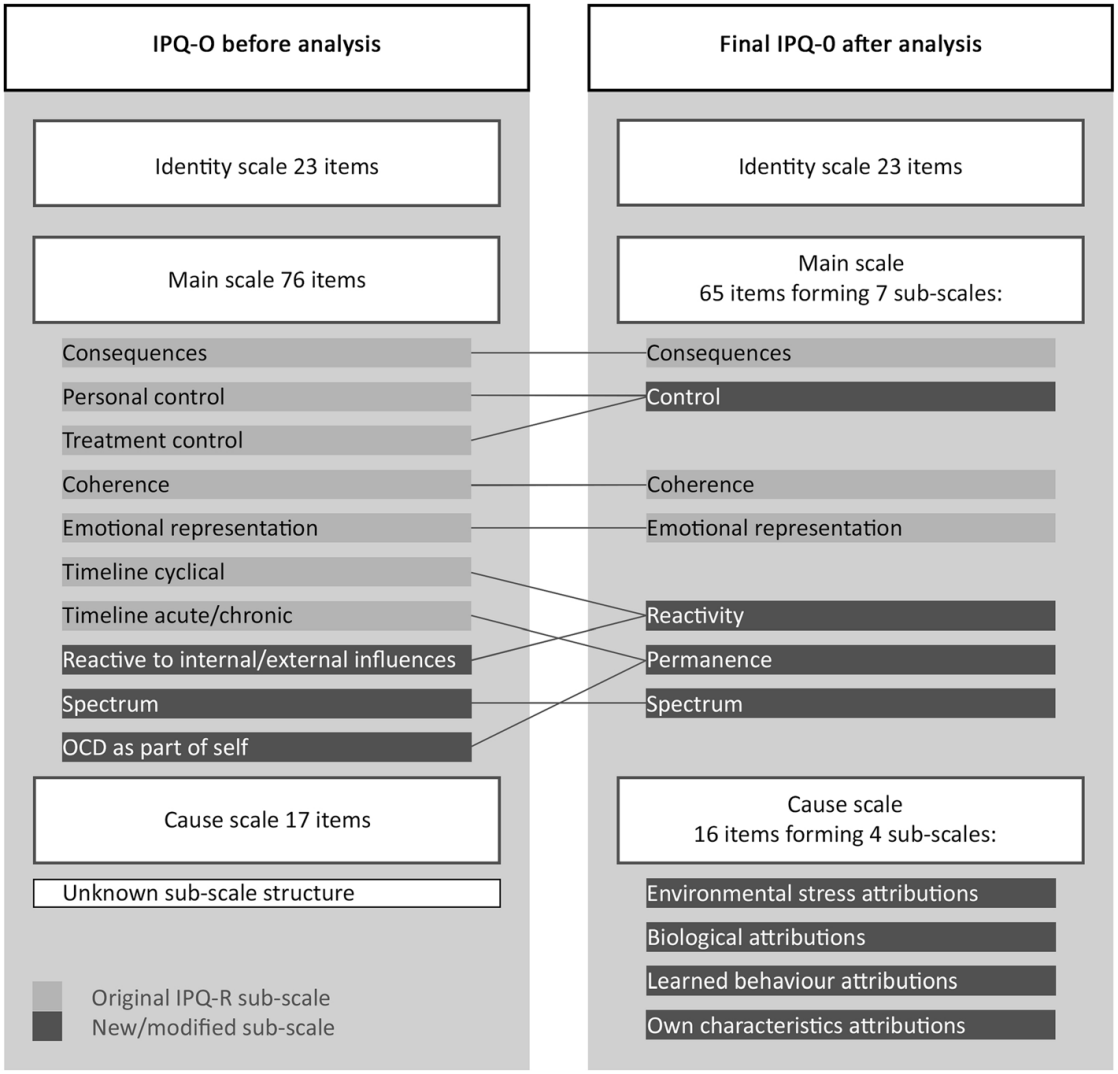
Diagram to illustrate number of IPQ-O items and proposed structure before and after testing and analysis

Figure 1 shows the structure and sub-scale content of the IPQ-O before and after analysis.

There was little missing data on the IPQ-O main scales and cause scale, with a range of 1–9 and 1–5 cases missing respectively. As the maximum number of missing cases for an item (i.e. 9) constituted just 2.6% of the total IPQ-O main scale sample, no items were removed based on missing data alone.

#### Main set sub-scales

The KMO measure of sampling adequacy for the overall scale was .872, classified as ‘meritorious’ according to Kaiser et al. [24]. All individual item KMO values exceeded the .5 minimum value criterion [23, 24].

Table 3 shows the process of item removal. Inspection of the correlation matrix revealed one item pair >.8 (OIP70 and OIP67). Following removal of OIP70, factor analysis was run on the remaining 75 items. Factor analysis using seven factors produced the most interpretable solution. Five items were removed due to having no loadings above .3. Following re-run of the factor analysis with the remaining 70 items, an additional two items were removed due to no loadings above .3. This resulted in a scale of 68 items. Finally, Cronbach’s α was run on the items remaining in each of the seven sub-scales, formed from the seven factors. Sub-scale 1 exceeded 15 items and was thus reduced by removing the two items that contributed the least to α. A further item was removed from factor 5 as it contributed no additional value to α. The final factor structure including the remaining 65 items, across seven sub-scales is presented in Table 4. Sub-scales considerably exceeded the minimum α value of .7 (Table 4) with the exception of the spectrum sub-scale, which scored .592.

**Table 3:** Stepwise approach to main scale item removal using exploratory factor analysis and Cronbach’s alpha

Question number	Item	Justification for removal
76 items IPQ-O		
OIP70	My OCD is likely to be permanent rather than temporary	Inspection of correlation matrix showed this item highly correlated (.825) with OIP67. OIP67 retained due to lower missing data.
75 items IPQ-O		
OIP14	My symptoms get worse when my daily routine needs to change^a^	No factor loadings above .3
OIP13	My symptoms are worse when I am in particular places^a^	No factor loadings above .3
OIP2	I find it hard to separate what is ‘me’ and what is my OCD^a^	No factor loadings above .3
OIP35	My OCD gets worse when I have more time^a^	No factor loadings above .3
OIP22	My OCD makes me obsessive about things in general, such as my hobbies and interests^a^	No factor loadings above .3
70 item IPQ-O		
OIP9	My symptoms of OCD are affected by my physical health^a^	No factor loadings above .3
OIP24	My OCD makes me feel guilty^a^	No factor loadings above .3
68 item IPQ-O		
	Factor 1: OIP48 – Other people’s behaviour can make my OCD worse^a^	Item reduction of consequences scale - α reduced .001 when removed.
	Factor 1: OIP10. My OCD gets worse when I need to conceal my symptoms^a^	Item reduction of consequences scale - α reduced .001 when removed.
	Factor 5: OIP26. Hearing or talking about OCD could lead to me picking up new symptoms^a^	No change in α if removed.
Final 65 items		

Items marked ^a^were new items, not included in the original IPQ-R

**Table 4:** Rotated main scale factor loadings from the pattern matrix

IPQ-O main-scale question	Original IPQ-R item (IPX) or new item (N)	1. Consequences	2. Control	3. Permanence	4. Coherence	5. Reactivity	6. Spectrum	7. Emotion
OIP73. My OCD can strongly affect the way others see me	IP9	**0.723**	− 0.037	0.002	− 0.069	− 0.093	0.138	− 0.054
OIP31. My OCD causes difficulties for those who are close to me	IP11	**0.723**	0.047	0.069	0.032	−0.011	− 0.020	− 0.163
OIP58. My OCD has a negative effect on my relationships with others	N	**0.709**	0.028	0.033	−0.099	0.008	− 0.026	−0.040
OIP40. My OCD negatively affects the way I act towards those close to me	N	**0.678**	−0.028	0.019	−0.131	0.125	0.039	−0.065
OIP3. My OCD strongly affects the way others act towards me	N	**0.671**	−0.126	−0.016	− 0.058	−0.094	0.203	−0.092
OIP75. My OCD has major consequences on my life	IP7	**0.640**	−0.063	0.078	0.028	−0.007	−0.060	0.168
OIP74. My OCD gets in the way of me getting things done	N	**0.550**	−0.123	0.007	0.032	0.044	−0.043	0.180
OIP56. My OCD affects my physical health and wellbeing	N	**0.505**	−0.024	0.001	0.028	0.142	−0.047	0.078
OIP11. My OCD has serious financial consequences	IP10	**0.501**	−0.090	−0.181	0.085	−0.066	0.061	0.211
OIP8. I have had difficulties with my work or studies because of my OCD	N	**0.466**	0.030	0.004	0.084	0.098	−0.162	0.159
OIP59. My OCD does not have much effect on my life (r)	IP8	**0.452** ^**a**^	− 0.029	**0.306**	0.041	−0.068	−0.143	0.162
OIP5. My OCD makes me feel worthless	N	**0.424**	−0.034	0.009	−0.157	− 0.030	0.049	0.264
OIP15. My OCD makes me feel ashamed or embarrassed	N	**0.364**	−0.033	− 0.001	− 0.224	0.051	0.008	0.225
OIP51. My OCD is a serious condition	IP6	**0.344**	0.070	0.210	0.055	0.043	−0.167	0.263
OIP54. Treatment will be effective if I put in enough effort	N	−0.040	**0.809**	0.049	−0.036	− 0.016	0.129	0.196
OIP71. Treatment can control my OCD	IP22	−0.043	**0.782**	−0.003	−0.012	0.015	0.065	0.106
OIP49. Treatment will be effective with a good health professional	N	−0.059	**0.736**	−0.023	− 0.044	0.077	0.063	0.170
OIP63. I have the power to influence my OCD	IP16	−0.095	**0.698**	0.072	0.088	−0.033	0.080	−0.022
OIP46. The negative effects of my OCD can be prevented (avoided) by my treatment	IP21	− 0.023	**0.656**	− 0.150	−0.059	0.002	0.133	0.111
OIP36. There is nothing which can help my condition (r)	IP23	−0.074	**0.654**	−0.054	0.132	0.038	−0.220	−0.132
OIP16. There is very little that can be done to improve my OCD (r)	IP19	−0.133	**0.637**	−0.100	0.102	−0.025	−0.127	− 0.123
OIP41. What I do can determine whether my OCD gets better or worse	IP13	0.075	**0.615**	−0.007	0.065	0.123	−0.011	−0.078
OIP39. There is a lot which I can do to control my OCD	IP12	−0.026	**0.615**	−0.047	0.128	0.008	0.162	− 0.091
OIP60. Nothing I do will affect my OCD (r)	IP15	0.053	**0.591**	0.047	0.025	0.019	−0.263	−0.170
OIP62. My OCD will improve in time (r)	IP18	0.057	**−0.571**	**0.303** ^**a**^	0.022	−0.033	0.029	−0.014
OIP33. Treatment will be effective in curing my OCD	IP20	−0.004	**0.561** ^**a**^	**−0.329**	0.010	−0.029	0.125	0.152
OIP61. My actions will have no effect on the outcome of my OCD (r)	IP17	0.003	**0.527**	0.128	0.101	−0.026	− 0.256	− 0.171
OIP30. The course of my OCD depends on me	IP14	0.020	**0.509**	−0.018	0.111	0.083	0.131	−0.053
OIP37. My OCD will last for a long time	IP3	0.069	−0.100	**0.747**	−0.008	−0.002	0.004	0.102
OIP67. I expect to have this OCD for the rest of my life	IP5	−0.022	−0.130	**0.733**	−0.002	0.066	0.064	−0.022
OIP44. I expect that some of my OCD symptoms will never go away	N	−0.016	−0.084	**0.717**	−0.018	0.043	0.072	−0.015
OIP1. My OCD will last a short time (r)	IP1	0.026	−0.064	**0.690**	0.057	−0.102	−0.185	0.070
OIP20. This OCD will pass quickly (r)	IP4	0.036	−0.113	**0.670**	0.016	−0.123	−0.144	0.044
OIP57. Looking back, I have always had OCD ‘traits’	N	0.037	0.155	**0.539**	−0.048	0.027	−0.019	−0.055
OIP42. Having OCD is part of my personality	N	−0.110	−0.030	**0.533** ^**a**^	−0.021	0.074	**0.401**	−0.033
OIP4. OCD has become part of who I am	N	0.081	−0.155	**0.459**	0.027	0.134	0.194	0.074
OIP17. I can’t remember how I felt when I didn’t have OCD	N	0.034	−0.130	**0.373**	−0.048	0.128	0.027	0.169
OIP55. I don’t understand my OCD (r)	IP26	0.053	0.042	−0.007	**0.858**	0.063	−0.038	0.005
OIP50. My OCD doesn’t make any sense to me (r)	IP27	0.027	0.009	0.026	**0.805**	0.079	−0.073	−0.118
OIP32. My OCD is a mystery to me (r)	IP25	0.070	0.038	−0.080	**0.782**	0.027	− 0.041	−0.055
OIP23.The symptoms of my condition are puzzling to me (r)	IP24	−0.031	−0.055	− 0.028	**0.716**	− 0.028	0.009	− 0.028
OIP28. I have a clear picture or understanding of my condition	IP28	−0.052	0.114	0.139	**0.590**	−0.001	0.084	0.202
OIP27. The types of OCD symptoms I have change depending on what is going on in my life at the time	N	−0.105	− 0.103	− 0.045	0.133	**0.740**	0.146	0.154
OIP38. My symptoms come and go in cycles	IP30	−0.177	0.092	−0.144	0.018	**0.680**	−0.018	−0.010
OIP47. The types of symptoms I have change over time	N	−0.101	0.075	0.016	0.024	**0.573**	0.148	0.172
OIP34. I go through cycles in which my OCD gets better and worse	IP32	−0.130	0.196	0.070	0.027	**0.559**	−0.077	0.016
OIP21. My symptoms of OCD are affected by my mood	N	0.054	0.037	0.059	−0.093	**0.491**	−0.026	− 0.168
OIP6. Old OCD symptoms reappear when I am tired	N	0.144	0.052	0.092	0.053	**0.473**	−0.119	− 0.152
OIP76. The symptoms of my OCD change a great deal from day to day	IP29	0.078	−0.056	− 0.089	− 0.086	**0.430**	0.212	0.025
OIP19. My symptoms get better when there are fewer pressures in my life	N	0.146	0.133	0.036	0.080	**0.402**	−0.019	−0.210
OIP72. I can find it difficult to tell whether or not OCD is affecting my thinking	N	−0.026	0.056	0.176	−0.256	**0.363**	−0.143	0.058
OIP66. My OCD gets worse when I take on new responsibilities	N	0.145	−0.021	0.045	0.010	**0.362**	0.011	0.048
OIP64. My OCD is very unpredictable	IP31	0.089	−0.024	0.039	−0.282	**0.359**	0.044	0.016
OIP25. Upsetting stories in the media can make my OCD worse	N	0.021	−0.064	0.048	−0.029	**0.336**	−0.229	0.146
OIP65. Everyone has a bit of OCD; it’s just that some people have more	N	0.054	0.001	−0.005	−0.045	− 0.049	**0.598**	− 0.052
OIP43. If my OCD was cured, it would change who I am as a person	N	0.103	−0.023	**0.367** ^**a**^	−0.041	− 0.005	**0.372**	0.112
OIP53. Everyone has compulsions to some extent	N	−0.054	0.097	0.224	−0.008	0.076	**0.362**	− 0.112
OIP18. People with OCD have the same worries as everyone else, just more extreme	N	0.035	0.167	− 0.068	−0.021	0.079	**0.362**	−0.008
OIP69. My OCD makes me feel ‘fed up’	N	0.186	−0.015	0.094	−0.195	0.034	−0.124	**0.625**
OIP68. When I think about my OCD I get upset	IP34	0.298	−0.037	0.027	−0.119	−0.016	− 0.078	**0.542**
OIP52. I get depressed when I think about my OCD	IP33	0.247	−0.076	0.168	−0.165	0.016	−0.054	**0.454**
OIP12. Having OCD makes me feel anxious	IP37	0.080	0.125	0.255	−0.079	0.080	0.010	**0.453**
OIP7. My OCD makes me feel afraid	IP38	0.242	0.103	−0.056	− 0.071	0.240	− 0.170	**0.415**
OIP29. My OCD does not worry me (r)	IP36	0.188	0.046	0.176	−0.079	−0.043	− 0.217	**0.411**
OIP45. My OCD makes me feel angry	IP35	**0.334**	−0.008	0.015	−0.120	0.124	−0.017	**0.366** ^**a**^
Total N questions		**14**	**13**	**11**	**5**	**12**	**3**	**7**
α		**0.893**	**0.914**	**0.857**	**0.867**	**0.789**	**0.592**	**0.860**

Loadings ≥ .3 are highlighted in bold

(r) represents reverse items

^a^Denotes which sub-scale a cross-loading item is allocated (where more than one loading ≥.3)

The seven sub-scales accounted for 45.5% of the variance after extraction and rotation. The sub-scales together with their associated variance accounted for after extraction/rotation were as follows: consequences (18.7%), control (9.5%), permanence (5.4%), coherence (4.7%), reactivity (2.8%), spectrum (2.5%), emotional representation (1.8%).

One of the three potential ‘new’ sub-scales, the ‘spectrum’ sub-scale (factor 6), loaded independently of the original IPQ-R scales. The other two potential new sub-scales (perceptions of OCD as part of the self and the internal/external factors that caused OCD to change) each loaded together with the timeline acute/chronic (re-titled ‘reactivity’) and the timeline cyclical sub-scales (re-titled ‘permanence’) respectively. The consequences sub-scale remained intact with the new consequences items loading as expected. Unexpectedly however, two new items measuring feeling ashamed/embarrassed and worthless, loaded together with consequences (OIP15 and OIP5). The personal control and treatment control sub-scales of the IPQ-R, loaded together on the same sub-scale, although supplemented by new control items as expected. The coherence sub-scale loadings contained all five original items from the IPQ-R. The emotional representation sub-scale remained intact, however supplemented by one new item relating to feeling ‘fed up’.

#### Cause scale

All items reached the minimum KMO criterion of .5 [23, 24]. The scree plot suggested that four factors should be extracted. Following factor analysis on the dataset (*n* = 331), one item was removed (‘A germ or a virus’) due to having no loadings above .3. The final analysis run with the remaining 16 items, together with each factor’s α can be viewed in Table 5.

**Table 5:** Rotated cause scale factor loadings from the pattern matrix

IPQ-O cause scale question	1. Environmental stress	2. Biological causes	3. Learned behaviour	4. Own characteristics or behaviour
OC8. Multiple stressful events	**0.812**	0.087	0.052	0.031
OC5. Experiencing traumatic events	**0.772**	−0.126	−0.007	0.122
OC13. A major change in my life	**0.615**	0.005	−0.001	−0.069
OC6. Relationship difficulties	**0.419**	−0.065	−0.146	− 0.175
OC7. Witnessing or hearing about something bad happening to someone else	**0.379**	0.08	−0.084	−0.112
OC11. Feeling that life was out of control	**0.376** ^**a**^	0.003	−0.171	**−0.302**
OC16. Lack of social or emotional support	**0.309**	−0.168	−0.207	− 0.264
OC2. The way in which my brain works or is wired	−0.118	**0.61**	0.039	−0.169
OC12. Inherited/caused by my genes	0.081	**0.584** ^**a**^	**−0.315**	0.193
OC1. A chemical or hormonal imbalance	0.031	**0.54**	0.1	−0.021
OC10. By learning from the behaviour of others in my family	−0.026	0.034	**−0.754**	−0.069
OC9. The way I was brought up or told to behave	0.119	−0.041	**−0.665**	− 0.018
OC14. My personality	−0.091	0.108	−0.123	**− 0.544**
OC4. Low self esteem	0.115	−0.048	0.038	**−0.531**
OC3. Unable to cope with stress very well	0.245	0.154	0.178	**−0.514**
OC15. A normal coping behaviour that got out of control	0.086	−0.183	−0.117	**− 0.427**
Total N questions	**7**	**3**	**2**	**4**
Cronbach’s alpha	**0.811**	**0.574**	**0.722**	**0.624**

Loadings ≥ .3 are highlighted in bold

^a^Denotes which sub-scale a cross-loading item is allocated (where more than one loading ≥.3)

The four factors accounted for 41.6% of variance after extraction. After extraction, the first factor ‘environmental stress’ accounted for the greatest amount of variance (24.3%), followed by the second factor, labelled ‘biological causes’ (7.5%), third factor ‘learned behaviour’ (5.9%) and lastly, fourth factor, ‘own characteristics or behaviour’ (3.9%).

Though the ‘learned behaviour’ sub-scale only contained two items, Cronbach’s α was satisfactory at .722 [23]. Cronbach’s α for the remaining variables was similarly adequate for the ‘environmental stress’ scale, but below recommended levels for the ‘biological’ and ‘own characteristics or behaviour’ scales. As all items were found to contribute to α and no sub-scales exceeded 15 items, it was not necessary to remove any additional items.

### Total sub-scale scores

Mean scores for each IPQ-O sub-scale are provided in Table 6.

**Table 6:** Descriptive statistics of total IPQ-O sub-scale scores

Scales (possible score range)	Mean item level sub-scale score (mean total sub-scale score / N items in sub-scale)	N^a^	Meaning of a high sub-scale score
Identity scale (0–23)	N/A	260^b^	Many symptoms attributed to OCD
Main sub-scales			
1. Consequences (14–70)	3.76	321	Many negative consequences caused by OCD
2. Control (13–65)	3.46	319	Positive beliefs about being able to control OCD
3. Permanence (11–55)	3.97	332	Perceiving OCD as permanent and therefore inseparable from self.
4. Coherence (5–25)	3.53	334	Positive views reflecting perceptions of a coherent understanding of OCD
5. Reactivity (12–60)	3.55	324	Perceptions that OCD symptoms are changeable/reactive
6. Spectrum (5–15)	2.87	339	Perception that OCD presents as a trait in the general population
7. Emotional representation (7–35)	4.04	334	Many negative emotions associated with having the condition.
Causes sub-scales			
1.Environmental stress (7–35)	3.22	320	Perceptions that life stresses contributed to development of OCD
2. Biological causes (5–15)	3.73	324	Perception that OCD was caused by biological factors
3. Learned behaviour (5–10)	2.91	327	Perception that OCD developed through learning
4. Own characteristics or behaviour (5–20)	3.26	321	Perception that own characteristics (E.g. personality) or behaviour are responsible for OCD

^a^N varies as participants with any missing data for a given sub-scale were excluded from the sum calculation

^b^N was further reduced due to removal of participants who missed > 2 items across either identity columns (‘since’ and OCD ‘related’ symptoms). This was to remove individuals who incorrectly scored one of the columns, instead of both

### Inter sub-scale correlations

The matrix of correlations can be viewed in Table 7. All sub-scales showed multiple significant associations with other sub-scales. The emotional representation sub-scale was strongly correlated with consequences and significantly related to all but one other (i.e. learned behaviour) sub-scale.

**Table 7:** Kendall’s tau (τ) IPQ-O inter sub-scale correlations

		1	2	3	4	5	6	7	8	9	10	11	12
1. Identity													
2. Consequences	τ	.23**											
	N	244											
3. Control	τ	.01	−.24**										
	N	246	305										
4. Permanence	τ	.14**	.24**	−.24**									
	N	254	313	313									
5 Coherence	τ	−.01	−.18**	.28**	−.09*								
	N	255	313	311	325								
6. Reactivity	τ	.14**	.09*	.11**	.11**	−.07							
	N	248	308	305	318	317							
7. Spectrum	τ	−.01	−.02	.06	0.02	−.04	0.05						
	N	256	317	315	330	331	322						
8. Emotional representation	τ	.20**	.51**	−.20**	.26**	−.22**	.15**	−.09*					
	N	252	314	313	324	326	317	329					
9. Environmental attributions	τ	.10*	.23**	−.01	−.00	−.03	.18**	.12**	.15**				
	N	244	303	299	311	310	305	316	310				
10. Biological attributions	τ	.13**	.11**	.03	.17**	−.08	.20**	−.02	.16**	−.06			
	N	244	304	301	314	314	307	320	314	316			
11. Learned behaviour attributions	τ	.03	.03	.11**	.00	.00	.17**	.15**	−0.06	.28**	.06		
	N	247	307	304	317	317	310	323	317	319	322		
12. Own characteristics attributions	τ	.11*	.13**	−.01	.15**	−.11**	.20**	.18**	.10*	.36**	−.02	.16**	
	N	241	301	298	310	311	304	316	313	312	316	318	

** *P* < 0.01 (2-tailed) * *P* < 0.05 level (2-tailed)

### Test re-test reliability

Two-week re-test reliabilities (Table 8) were satisfactory, ranging from τ **=** .51 (spectrum) to τ = .75 (control), and all sub-scales exceeding the minimum (converted) reliability correlation value of τ **=** .49.

**Table 8:** Two-week test-retest reliabilities using Kendall’s tau (τ)

	τ	N
1. Identity	.67**	52
2. Consequences	.68**	58
3. Control	.75**	53
4. Permanence	.73**	62
5 Coherence	.68**	62
6. Reactivity	.69**	60
7. Spectrum	.51**	62
8. Emotional representation	.70**	62
9. Environmental attributions	.61**	61
10. Biological attributions	.61**	63
11. Learned behaviour attributions	.64**	64
12. Own characteristics attributions	.68**	60

** *P* < 0.01 (2-tailed)

### Construct validity

Based on previous literature and theoretical rationale, predictions were made about the expected relationships between IPQ-O sub-scales and continuous and categorical measures of emotional adjustment, treatment use and help-seeking plans (Table 9).

**Table 9:** Predicted associations between IPQ-O and other measures to assess construct validity

	Predicted associations with IPQ-O sub-scales
Continuous variables -	
OCD severity (Y-BOCS)	*Positively* correlated with identity, consequences and permanence.
Depression (PHQ-9)	*Positively* correlated with: identity, consequences, permanence, emotional representation and own characteristics attributions. *Negatively* correlated with control and coherence.
Anxiety (GAD-7)	*Positively* correlated with: identity, consequences, permanence, emotional representation. *Negatively* correlated with coherence.
Attitudes towards seeking mental health services (IASMHS)	IASMHS Openess sub-scale*: Positively* correlated with coherence. IASMHS Indifference to stigma sub-scale: *Positively* correlated with coherence (greater indifference to stigma associated with better coherence) and spectrum beliefs (beliefs that OCD is on a spectrum will be associated with less concern about stigma)
Functioning (WSAS)	*Positively* correlated with consequences. *Negatively* correlated with control.
Categorical variables - condition management	
No previous treatment for OCD (yes/no)	People who have low perceptions of control and permanence or perceive OCD as a spectrum condition would be less likely to have received treatment for OCD.
Received talking therapy for OCD (yes/no)	People who have received previous talking therapy for OCD will have significantly greater scores on the control sub-scale.
Received medication for OCD (yes/no)	People who have received medication for OCD will have significantly greater scores on the permanence sub-scales and lower scores on the spectrum sub-scale
Plans to seek help in future (yes/no) - NHS *Including: NHS General Practitioner, Self-referral to mental health services* e.g. *Improving Access to Psychological Therapies services (IAPT)*	People who plan to seek NHS help in the future will score more highly on the control and permanence sub-scales. People who plan to seek help will score lower on the spectrum sub-scale.
Plans to seek help in future (yes/no) - ‘other’ *Including: charity organisations, support groups, internet forums, use of self-help books*	People who plan to seek ‘other’ help in the future will score more highly on the control and permanence sub-scales. People who plan to seek help will score lower on the spectrum sub-scale.
Plans to seek help in future (yes/no) - private therapy	People who plan to seek private therapy in the future will score more highly on the control and permanence sub-scales. People who plan to seek help will score lower on the spectrum sub-scale.

Table 10 presents correlations between IPQ-O sub-scales and the other assessed variables.

**Table 10:** Kendall’s tau (τ) correlations between IPQ-O sub-scales and other continuous scales to assess construct validity

IPQ-O sub-scale		OCD severity (Y-BOCS total)	OCD severity (Y-BOCS obsession total)	OCD severity (Y-BOCS compulsion total)	Depression (PHQ-9 total)	Anxiety (GAD total)	Attitudes towards seeking mental health services – Openness (IASMHS)	Attitudes towards seeking mental health services - Help seeking propensity (IASMHS)	Attitudes towards seeking mental health services - Indifference stigma (IASMHS)	Functioning (WSAS^a^)
1 Identity	τ	.15**	.18**	.11*	.15**	.22**	.08	−.02	0	.110
	N	234	237	237	230	228	224	226	224	110
2.Consequences	τ	.45**	.43**	.41**	.39**	.39**	−.09*	−.18**	−.27**	.47**
	N	292	296	294	280	278	272	277	275	137
3.Control	τ	−.38**	−.32**	−.37**	−.29**	−.24**	.08	.32**	.17**	−.26**
	N	289	293	291	277	276	272	274	272	139
4.Permanence	τ	.23**	.18**	.24**	.21**	.24**	−.02	−.12**	−.13**	.09
	N	300	304	303	288	288	280	285	283	140
5.Coherence	τ	−.16**	−.13**	−.15**	−.15**	−.12**	.18**	.08	.24**	−.11
	N	302	305	305	288	289	282	287	284	140
6.Reactivity	τ	−.03	−.01	−.05	.07	.11*	.00	.04	−.04	.07
	N	294	298	297	283	283	275	280	279	141
7.Spectrum	τ	−.13**	−.12**	−.13**	−.14**	−.13**	−.06	.16**	.01	−.16**
	N	307	311	310	294	295	287	291	289	145
8.Emotional representation	τ	.37**	.38**	.33**	.29**	.38**	−.07	−.08	−.20**	.29**
	N	301	305	303	288	288	282	288	284	142
9.Environmental stress attributions	τ	.09*	.12**	.07	.13**	.13**	−.03	.09*	−.09*	.17**
	N	301	305	304	289	288	282	286	284	145
10.Biological attributions	τ	.05	.05	.04	.06	.07	.02	.05	−.05	.06
	N	305	309	308	292	292	285	289	287	145
11.Learned behaviour attributions	τ	−.07	−.03	−.09*	−.03	−.06	.01	.10*	−.07	.03
	N	308	312	311	295	295	288	292	290	146
12.Own characteristics or behaviour attributions	τ	.06	.06	.05	.16**	.12**	−.13**	.03	−.08	.03
	N	303	307	306	290	290	284	288	286	143

** *P* < 0.01 (2-tailed) * *P* < 0.05 level (2-tailed)

^a^Ns for the WSAS scale are smaller than other measures due to error in how the WSAS scale was displayed to early completers of the online measure. Only participants taking part after the online questionnaire error was corrected are included for this scale

#### Depression and anxiety

As predicted, higher levels of depression (PHQ-9) and anxiety (GAD-7) were significantly associated with a stronger illness identity, higher levels of negative consequences, greater illness permanence and a stronger emotional representation. Also as predicted, there were significant negative associations between coherence and depression and anxiety, as well as between perceptions of control and participants’ depression scores. Perceptions of OCD as caused by one’s own characteristics or behaviour were associated with higher depression scores. Correlations between anxiety, depression and the consequences sub-scale were large (**τ =** .39).

#### OCD severity

Significant positive correlations were found between OCD symptom severity and participants’ illness identity, consequences and permanence. Correlations with illness permanence and consequences were moderate (τ = .23) and large respectively (τ **= .**45).

#### Functioning

Participant functioning (WSAS) was significantly positively associated with perceived consequences (large association, τ = .47), and negatively associated with control (medium association, τ = −.26).

#### Attitudes towards seeking mental health services

As expected, participants who had a more coherent understanding of their OCD scored higher (more positive attitudes) on IASMHS sub-scales psychological openness (small association, τ = .18) and indifference to stigma (medium association, τ = .24).

Our prediction that people who believed OCD to be a spectrum in the general population would be associated with indifference to stigma was not supported (τ non-significant).

### Relationships with condition management variables

The results of the Mann-Whitney U tests (two-tailed), testing predictions about relationships between IPQ-O sub-scales and treatments received and help-seeking intentions, are presented in Table 11.

**Table 11:** Mann Whitney U tests testing predicted group differences in help-seeking intentions according to IPQ-O dimensions

IPQ-O sub-scale		*N*	*Mdn*	*U*	*Z*	*P*
Control	Received talking therapy for OCD (CBT/other)	279	46	4140.000	−2.641	.008**
	Not received previous talking therapy for OCD	40	42			
	Planning to seek NHS help in future	47	44	6176.500	−.369	.712
	Not planning to seek NHS help in the future	272	46			
	Planning to seek ‘other’ help in the future	95	46	10,253.500	−.513	.608
	Not planning to seek ‘other’ help in the future	224	45			
	Planning to seek ‘private’ help future	35	46	4520.500	−.874	.382
	Not planning to seek ‘private’ help future	284	45			
Spectrum	Received medication for OCD	244	9	9928.500	−2.065	.039*
	Not received medication for OCD	95	9			
	Planning to seek NHS help in future	49	9	6584.500	−.826	.409
	Not planning to seek NHS help in the future	290	9			
	Planning to seek ‘other’ help in the future	100	9	11,937.000	−.016	.987
	Not planning to seek ‘other’ help in the future	239	9			
	Planning to seek ‘private’ help future	36	8	4376.000	−1.953	.051
	Not planning to seek ‘private’ help future	303	9			
Permanence	Received medication for OCD	240	45	9543.500	−1.914	.056
	Not received medication for OCD	92	43			
	Planning to seek NHS help in future	47	48	5126.500	−2.580	.010*
	Not planning to seek NHS help in the future	285	44			
	Planning to seek ‘other’ help in the future	97	45	11,278.000	−.150	.880
	Not planning to seek ‘other’ help in the future	235	44			
	Planning to seek ‘private’ help future	35	46	4645.500	−1.029	.304
	Not planning to seek ‘private’ help future	297	44			

** *P* < 0.01 (2-tailed) * *P* < 0.05 level (2-tailed)

Few individuals (see Table 1) had received ‘no’ treatment for OCD, so we did not assess the associations between illness perceptions and receiving no vs any treatment.

#### Control

As predicted, those who had received talking therapy perceived significantly more control (IPQ-O ‘control’ sub-scale) over their OCD than those who had not received talking therapy. Inconsistent with predictions, there were no significant differences in control beliefs between those who did and *did not* plan to seek future help (NHS help, ‘other’ help, private therapy).

#### Spectrum

As predicted, those who received medication for OCD, had significantly lower scores on the spectrum sub-scale than those who had not. However, there was no association between spectrum beliefs and planning or not planning to seek any modality of help (Table 10).

#### Permanence

As predicted, those who saw OCD as more permanent were significantly more likely to plan to seek NHS help in the future.

## Discussion

Psychometric testing has enabled the development of a reliable and valid measure of illness perceptions, adapted for OCD. Mirroring the format of the IPQ-R, the IPQ-O is made up of three sections: first, the identity scale, which reflects OCD specific symptoms discussed by people with OCD as part of qualitative interviews, second; the main-subscales, consisting of seven dimensions derived from factor analysis and thirdly, the cause sub-scales, made up of four sub-scales identified through factor analysis that represent the views of people with OCD.

The resultant set of main sub-scales included three of the original IPQ-R sub-scales. These three sub-scales (consequences, coherence and the emotional representation) are now validated in an OCD sample. The coherence scale includes all original IPQ-R items, however, the other two sub-scales were made more specific to OCD through the addition of new items. Eight new items supplemented the consequences sub-scale. Amongst these, the strongest loading items reflect negative social consequences of OCD. Two of these items were originally expected to fit with emotional representation items (worthlessness, shame/embarrassment). It is possible that these emotions load together with consequence items as they incorporate elements of social concerns/evaluation to a greater extent than the other emotional representation items (e.g. being afraid, upset, anxious etc.). The emphasis on social impairment within the OCD-specific consequences sub-scale is consistent with the literature. The social domain of quality of life in OCD has been shown to be particularly reduced compared with other mental health or physical illnesses [29]. The retention of an independent emotional representation dimension (supplemented by one ‘new’ item) is noteworthy as it supports the conceptual difference between cognitive and emotional representations proposed by the CSM [4].

Despite support for three of the IPQ-R’s dimensions, the remaining sub-scales highlighted differences in the way people with OCD perceive their condition compared with chronic physical health conditions. Three potential new sub-scales were derived from our previous qualitative study [13]. One of these sub-scales, perceptions of OCD as on a spectrum in the general population, was validated as a distinct sub-scale. Items from the other two proposed sub-scales combined with items from existing IPQ-R dimensions to create new meaning. One of these sub-scales, perceptions of OCD as part of the self, loaded with timeline acute/chronic items, a scale which in the original IPQ-R measures respondents’ beliefs about the illnesses’ chronicity [2]. Though these dimensions were discussed separately within our qualitative findings, the loading together of these items is understandable as the perceived permanence of OCD contributed to participants’ perceptions of the condition as ‘part’ of themselves [13]. There was a sense that OCD was now ‘ingrained’ and that even with treatment, it would only improve but never fully remit. Thus, OCD was accepted as a permanent part of self-identity. We would posit that these items load together because acceptance of OCD as part of the self is an extreme view of illness permanence. Notably, the mean sub-scale score for permanence items was 3.97, demonstrating that the sample endorsed a perception of the condition as permanent, consistent with previous qualitative findings [13]. Additionally, timeline cyclical items (original IPQ-R sub-scale, describing the extent to which respondents perceive the illness as fluctuating over time) loaded together with the ‘reactive’ items. This was not entirely unexpected as we had previously theorised that these dimensions may overlap [13]. We propose that the new sub-scale now more fully captures the extent to which people see OCD as changing and reacting to internal (e.g. mood) and environmental influences (e.g. life pressures).

The final dimension from the set of main-scales was formed through the combination of the personal control and treatment control sub-scales, creating a general ‘control’ sub-scale. Though Moss-Morris et al. [4] retained both these sub-scales separately in the IPQ-R, they nevertheless noted cross-loading between the two and emphasised that the importance of their distinction may vary depending on the condition assessed. Our previous qualitative study suggested that participants’ sense of greater personal control resulted from skills learned through psychological therapies, such as abstaining from performing rituals [13]. Perhaps in the case of our sample, the majority of whom had received previous cognitive-behavioural therapy (CBT) for OCD, these constructs are too inter-dependent to be distinguishable. This raises a broader point about whether these two control aspects are more closely aligned in mental health (as opposed to physical health) due to the reliance in therapies such as CBT, on behavioural changes made by the individual [30].

The cause scale largely consists of items generated through the qualitative interviews and thus closely reflects the perceptions of people with OCD. Factor analysis of the resulting scale led to the identification of four sub-scales, representing environmental stress, biological causes, learned behaviour and own characteristics or behaviour.

The main sub-scales were found to be internally consistent, with the exception of the spectrum sub-scale (α .592), which only had three items; fewer items are known to be associated with lower α values [23]. Two of the cause sub-scales also fell below the .7 criterion. It has been suggested that, when developing new scales, an alpha of .5 is sufficient to warrant further scale development [26]. As this was the first attempt to measure these constructs (spectrum, biological causes and own characteristic causes) in OCD, these values could be argued to be acceptable, however future iterations should seek to improve them.

Due to the unavailability of criteria for interpreting τ correlations, we used equivalent criterion values for small, medium and large correlations, as well as the minimum test-re-test value, using a table for converting r to τ. All sub-scales exceeded the minimum equivalent value of r > .7 (τ > .49) for test re-test reliability, suggesting that the IPQ-O is reliable. Although within acceptable limits when using Kendall’s τau, the weaker performance of the spectrum sub-scale scale may again be due to the low number of items in this sub-scale. With so few items, small changes in item scores over time would have made a greater relative difference to total score values compared to sub-scales with a larger number of items. Additional items could be added to the spectrum scale in a future iteration of the measure to strengthen this sub-scale’s test-re-test reliability, alongside its internal consistency.

The pattern of correlations between IPQ-O sub-scales and other scales which were expected to be theoretically linked, generally supported the validity of the IPQ-O sub-scales. For example, a stronger illness identity, believing OCD to be permanent, having many negative consequences and a strong emotional representation, were associated with higher levels of depression and anxiety. Conversely, perceptions of having control over OCD was associated with better functioning and lower depression.

As the CSM suggests that perceptions of illness should drive behaviours to deal with the illness, including help-seeking behaviours, we were interested to see whether previous therapies received and intention to seek further therapy were associated with the IPQ-O illness perception dimensions. Those who had received talking therapy perceived greater control over their OCD than those who had not. Since learning skills to self-manage problems is a key goal of talking therapies such as CBT [31], this finding makes conceptual sense, and is in line with the CSM which holds that the results of actions (such as therapy seeking) are appraised, and the results of that appraisal fed back into the model, potentially modifying it [1]. People who received medication for OCD had lower scores on the spectrum sub-scale. It seems plausible that people who perceive OCD as a ‘trait’ present to differing extents throughout the population (as opposed to an illness) would reject a ‘medicalised’ view of OCD as an ‘illness’ and be less willing to take medication as a result. This is consistent with findings of an OCD treatment preference study, which showed that some people do not view medication as appropriate treatment for ‘psychological’ problems [32]. Though inferences about cause and effect cannot be derived from this analysis, further longitudinal research which tests this premise is warranted, as if linked in the posited way, such beliefs could influence the uptake of potentially efficacious treatments such as selective serotonin reuptake inhibitors (SSRIs) [33]. Although differences between perceptions of OCD permanence between those who had and had not taken medication for OCD were not significant, findings were close to significance, with a higher level of perceived permanence in those who had received medication.

We could not assess whether there was a relationship between those who had received no previous treatment versus having received any treatment and IPQ-O sub-scales, as so few individuals had received *no* previous OCD treatment. This suggests that our sample may be biased towards individuals who seek help and engage in treatment. Our recruitment methods, which relied heavily on advertising via OCD charities mean that we are likely to have recruited individuals who were already engaging in help seeking and therefore, were more likely to have received treatment. Though there is a known gap between symptom onset and treatment [10], recruiting treatment naïve individuals who have a diagnosis of OCD is likely to be difficult as diagnosis is likely to be given at the point of treatment access.

As expected, people who planned to seek NHS treatment (e.g. GP, IAPT services) saw OCD as more permanent. This prediction was based on previous findings that expectations of a long timeline in other conditions were associated with treatment use [34]. However, an alternative prediction could have been made based on the ‘new’ meaning of our sub-scale, that perceptions of OCD as permanent part of the self might lead to individuals to perceive treatment as futile and therefore not worth seeking. Our qualitative findings, however, showed that despite participants having received psychological therapy for OCD, most nevertheless saw the condition as permanent [13]. Although many thought treatments had improved or could improve their condition, most remained doubtful that treatment could ‘cure’ their OCD. This suggests that individuals may still be willing to seek treatment despite doubts about its curability. A better question may therefore not be whether individuals plan to seek therapy, but whether perceptions of permanence impact on outcomes of psychological therapy, as pessimism could impede progress made during treatment e.g. engagement in exposure exercises. Future studies could investigate this by administering the IPQ-O at the start of treatment and examining whether perceptions predict outcome.

Spectrum beliefs were negatively associated with OCD severity, functional impairment, depression and anxiety severity. This suggests that people who have less severe problems are more likely to see OCD as a trait in the general population. It could reflect a possibility that people who ‘normalise’ OCD as something everyone has to differing extents, are less distressed.

### Limitations

We did not verify diagnosis of OCD through any diagnostic tool, making it possible that some participants did not have current OCD. The decision not to use a diagnostic tool was due to the practical difficulties in administering this to individuals participating remotely, without the presence of a researcher. Remote completion also meant that there was less control over the particular settings in which individuals participated, compared with completion supervised by a researcher. We believe these limitations were however outweighed by the advantages of remote completion. Remote completion did not restrict participation to individuals within geographical reach of the researcher and allowed for anonymous participation, which we hoped would increase the diversity and size of the sample. Our recruitment of participants already engaging in appropriate channels (OCD/anxiety charities), the majority of whom had already received treatment for OCD and who self-reported receipt of an OCD diagnosis, gives some confidence that our sample reflects the appropriate population. Future studies would, however, benefit from including a more robust verification of OCD diagnosis, using a validated tool.

It is also important to note the considerable time that participants had experienced OCD symptoms at the point of interview (M: 18 years) and that three quarters of the sample were female, whereas OCD occurs in approximately equal proportions in males and females worldwide [35]. As it is possible that gender and the length of time experiencing OCD could affect illness perceptions, future studies that use the IPQ-O to investigate how perceptions impact on outcomes should seek to recruit more representative samples, for example, by recruiting individuals as they enter treatment.

Finally, in line with the IPQ-R and our preliminary qualitative work [13], our measure has been validated in an adult sample (age 16+) and cannot currently be used in a child/adolescent sample. It may also be useful to develop and test an illness perceptions measure for young people; particularly given that OCD symptom onset is frequently during childhood/adolescence [8].

## Conclusions

Recruitment of a large sample has made it possible to employ factor analysis to derive a modified OCD-specific questionnaire, capable of capturing the way people understand the condition. Development of the IPQ-O using qualitative data has led to the identification of types of illness perception not previously assessed in other conditions. Of particular significance, the spectrum scale could be considered an ‘anti-illness’ dimension of the IPQ-O, which measures perceptions of the condition as a ‘trait’. The measure demonstrates internal consistency, test-re-test reliability and convergent validity. Future studies should test the identified factor structure of the measure using a new sample, through confirmatory factor analysis [24].

The current study has demonstrated that perceptions of OCD are associated with important aspects of adjustment and treatment behaviours (use of talking therapies and medication). Though help-seeking plans were less reliably associated, this was possibly due to the majority having already engaged in treatment, including talking therapies. Longitudinal studies are now needed to test whether the identified perceptions drive outcomes; for example, by examining whether baseline illness perceptions drive outcomes after treatment. If illness perceptions drive behaviours and adjustment to OCD as hypothesised by the CSM, this would indicate a need to address perceptions that negatively affect coping behaviours and adjustment as part of future interventions. For example, if perceptions of OCD on a spectrum hinder use of medication for OCD, health professionals might seek to challenge this perception as part of individual therapy, as well as through future initiatives (e.g. in primary care) that promote help seeking and treatment use in OCD. The IPQ-O offers a reliable and valid tool to assess perceptions of OCD in clinical and research settings alike.

## Data Availability

The (anonymised) datasets analysed during the current study are available from the corresponding author on reasonable request.

## Abbreviations

CBT: Cognitive-behavioural therapy
EM: Expectation-maximisation
GAD-7: Generalised Anxiety Disorder 7
IAPT: Improving Access to Psychological Therapies programme
IASMHS: Inventory of Attitudes toward Seeking Mental Health Services
IPQ-O: Illness perceptions questionnaire for OCD
IPQ-R: Illness perceptions questionnaire-revised
KMO: Kaiser-Meyer-Olkin
MCAR: Missing completely at random
OCD: Obsessive-compulsive disorder
PHQ-9: Patient Health Questionnaire
WSAS: Work and Social Adjustment scale
Y-BOCS: Yale-Brown Obsessive Compulsive Scale
